# Commemorating Toxicology at the National Institute of Environmental Health Sciences on the Occasion of Its 50th Anniversary

**DOI:** 10.1289/EHP463

**Published:** 2016-11-01

**Authors:** John R. Bucher, Linda S. Birnbaum

**Affiliations:** 1 Division of the National Toxicology Program (NTP), and; 2 National Institute of Environmental Health Sciences (NIEHS), National Institutes of Health, Department of Health and Human Services, Research Triangle Park, North Carolina USA

## Abstract

In 1978, the National Toxicology Program (NTP) was established and headquartered at the National Institute of Environmental Health Sciences (NIEHS) in Research Triangle Park, North Carolina. On the occasion of the 50th Anniversary of the NIEHS, this article documents some of the historical and current NTP programs and scientific advances that have been made possible through this long-standing relationship.

## Genesis of the NTP

In the history of modern toxicology, few years have been more consequential than 1978—the year the National Toxicology Program (NTP) came into being. On 7 August 1978, following months of reports of serious health problems near a chemical dump site at Love Canal, New York, former President Jimmy Carter declared a federal health emergency to facilitate clean up and the relocation of nearby families. On November 6, the Congress passed an amendment to the Public Health Service Act of 1944 (U.S. DHHS 2011) mandating, among other things, the regular publication of a Report on Carcinogens: Six days later, the U.S. Department of Health, Education and Welfare [now the U.S. Department of Health and Human Services (DHHS)] Secretary Joseph Califano established the NTP under the umbrella of the National Institute of Environmental Health Sciences (NIEHS). At the time, the NTP was the only comprehensive toxicology-testing program in the world.

The NIEHS Director, David Rall, was the driving force behind the inception and creation of the NTP. He envisioned an independent program that transcended the regulated community (Huff 2005) as well as individual regulatory agencies to provide a broad, credible approach to toxicology. To that end, he succeeded in bringing into the NTP components from NIEHS, the National Cancer Institute, the Food and Drug Administration’s National Center for Toxicological Research (NCTR), and the National Institute for Occupational Safety and Health (NIOSH) at the Centers for Disease Control (now the Centers for Disease Control and Prevention). Together with the first NTP Associate Director, John Moore, Rall oversaw the transfer of the National Cancer Institute Rodent Cancer Bioassay Program to the NTP, which became the cornerstone of a comprehensive chemical toxicology characterization program.

## Public Interest Goals

Among NTP’s mandates under the amended Public Health Service Act of 1944 was the periodic preparation of the “Report on Carcinogens” (RoC), the first of which appeared in July 1980. The 26 chemicals or industrial processes in the report that were known human carcinogens constituted the first such official government list of its kind. The 13th edition of the RoC, released in 2014 by the Secretary of the U.S. DHHS (http://ntp.niehs.nih.gov/pubhealth/roc/roc13/index.html), profiles 243 substances or classes of chemicals that are now listed as known or reasonably anticipated to be human carcinogens. Although some listings have proven to be controversial over the years, listing decisions have withstood challenges in the courts and have been affirmed by the National Academy of Sciences, attesting to the scientific rigor of the listing review process.

**Figure 1:**
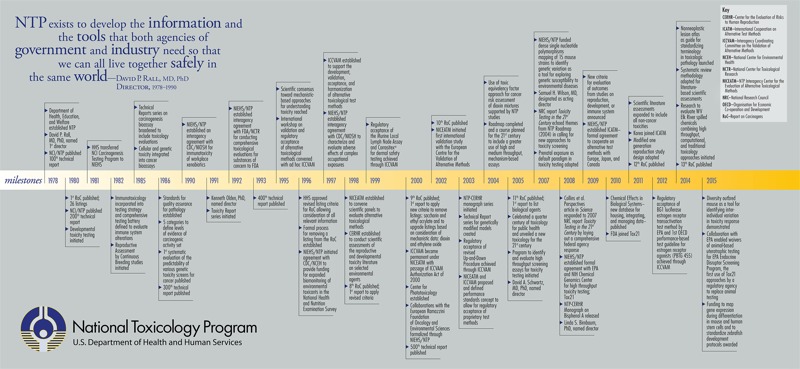
Flowchart highlighting the milestones of the National Toxicology Program, 1978–2015.

As the focal point for coordinating toxicology research and the testing of chemical and physical agents in the environment, the NTP’s commitment from the beginning has been to strengthen the science of toxicology, develop and validate improved testing methods, and provide information about potentially toxic substances to health regulatory and research agencies, the scientific and medical communities, and the public (Figure 1).

That commitment, especially to the public, has often made the NTP a go-to agency during public health emergencies, at home and around the world. Examples include the chemical leak in Bhopal, India, in 1983, where NTP staff conducted research on the long-term health effects of methyl isocyanate. In 1985, after a suspicious dust was found in the U.S. Embassy in Moscow, NTP scientists were called upon to assure American diplomats that their health had not been compromised. And almost immediately after the terrorist attacks of 11 September 2001, NTP studied dust samples to gauge the long-term health effects on New York City emergency responders. Other examples of substances of particular public interest about which NTP provided critical research findings include AZT (azidothymidine) and a number of drug combinations used to prevent mother-to-child transmission of HIV (human immunodeficiency virus); fluoride; polychlorinated biphenyls; hexavalent chromium; acrylamide; silicone fluid used in breast implants; and phenolphthalein, which was once used in laxatives.

## New Methods, Ongoing Concerns

In addition to carcinogenesis, the NTP’s particular emphasis on rigorous standardization of testing methodologies for genetic toxicology, reproductive and developmental toxicology, and immunotoxicology has resulted in many of its testing protocols and evaluation criteria being adopted and adapted for use worldwide (Chhabra et al. 2003; Luster et al. 1988; Tilson 1990). The agency has also been a leader in evaluating and incorporating into its research and testing programs many new technologies, including (with a representative reference) mouse models with oncogene or suppressor gene mutations for more rapid cancer screening (Pritchard et al. 2003), digital pathology imaging of lesions (Cesta et al. 2014), statistical approaches for data analysis (Haseman 1984), molecular analyses of oncogene and tumor suppressor gene mutations (Ton et al. 2004), measurements of global and specific gene expression changes (Hoenerhoff et al. 2011), mouse models with wide genetic diversity (French et al. 2015), and a modified one-generation reproduction and development testing protocol (Foster 2014).

Helping regulatory agencies develop alternatives to traditional animal-based toxicology testing has also been an NTP focus. In 1997, the 15-member Interagency Coordinating Committee on the Validation of Alternative Methods (ICCVAM) was established. With NTP support, ICCVAM has evaluated numerous assays that reduce, refine, or replace the use of animals in required toxicology screens and has led a worldwide effort, in partnership with international agencies with similar goals, to comply with legislative initiatives that limit or prohibit animal-based safety assessments (Casey et al. 2015).

As NTP methodology has progressed hand in hand with strong research and testing, more than 2,800 substances have been studied, including industrial chemicals, drugs, botanicals, metals, nanoscale materials, water disinfection by-products, food contaminants, pyrolysis products, endocrine-active agents, and even electromagnetic fields. These studies have also addressed other important issues, such as the assumptions used to estimate cumulative risks from mixtures of persistent toxic chemicals (Walker et al. 2005) and specific vulnerabilities of early-life exposures (Chhabra et al. 1993a, 1993b; Delclos et al. 2001; Waalkes et al. 2014). In 1999, the NTP convened expert panels to prepare consensus reports on the risks to children’s health and development posed by exposure to environmental hazards, patterned after a private sector effort by the Institute for Evaluating Health Risks (Moore et al. 1995). In 2011, the NTP expanded these assessments to include non-cancer health hazards (Bucher et al. 2011), recognizing that many substances, including, for example, endocrine-active agents, can affect multiple biological processes. In 2008, the NTP published a monograph by the Center for the Evaluation of Risks to Human Reproduction (CERHR) on bisphenol A, expressing some concern over adverse effects on the brain, behavior, and prostate glands in fetuses, infants, and children (NTP 2008). This work built on an extensive body of academic research, much of which was funded through the NIEHS Division of Extramural Research and Training, and important earlier work of the NIEHS Division of Intramural Research, which established mouse models of human health effects of diethylstilbestrol (Newbold 2004).

### Tox21

In 2004, the NTP published “The National Toxicology Program for the 21st Century,” a roadmap for a new vision of the NTP: Its stated purpose is “To support the evolution of toxicology from a predominantly observational science at the level of disease-specific models to a predominantly predictive science focused upon a broad inclusion of target-specific, mechanism-based biological observations.” This led to the collaborative interagency effort, known as Tox21 (Toxicology in the 21st Century), that is fueling a revolution in toxicology and providing the conceptual and molecular basis for integrating and understanding toxicity in the broader context of environmentally induced disease and dysfunction (Collins et al. 2008; Tice et al. 2013).

### Meeting New Challenges

Two of the NTP’s most ambitious and technically challenging projects are now underway:

Radiofrequency radiation cancer studies are being carried out under contract at the Illinois Institute of Technology Research Institute (IITRI) in Chicago. Initial findings appear to support the International Agency for Research on Cancer conclusions that RFR is a possible human carcinogen (Wyde et al. 2016). These studies represent a complex technical collaboration between NTP, IITRI, the National Institute of Standards and Technology, and the Foundation for Research on Information Technologies in Zurich.
Comprehensive perinatal exposure toxicity and carcinogenicity studies of bisphenol A are being conducted through an interagency agreement at the National Center for Toxicological Research. The agreement provides tissues and animals to 13 NIEHS grantees attempting to reconcile discrepant views of the potential for this ubiquitous endocrine-active agent to adversely affect health during early-life exposure (Heindel et al. 2015). Studies of exposure to endocrine-active agents during all life stages exemplify the efforts of the NTP to bring the most advanced technological and scientific approaches to bear on topical problems of potentially huge public health importance.

The NTP is also bringing systematic review methodology, long employed in clinical medicine, into the study of environmental health. The intent is to improve the transparency and consistency of evaluations of literature used in reaching public health decisions (Birnbaum et al. 2013; Rooney et al. 2014). As with Tox21 and the alternative methods discussed above, the NTP is providing leadership in a worldwide effort to improve objectivity and clarity in public health decision-making.

The NTP has benefited greatly from its long association with NIEHS, through direct financial support and the close association between NTP’s staff and NIEHS’s intramural and extramural colleagues. In addition, we believe the problem-solving mission of the NTP has provided a practical influence and focus to the basic research activities of the intramural and grant programs. We wish to acknowledge all of the staff who have worked to further the goals of the NTP within NIEHS, at NIOSH, and at NCTR, and our colleagues at sister agencies who have participated in our activities over the years.

As the impact of the environment on human health becomes more fully appreciated (Wu et al. 2016), it is our firm belief that the NTP and the NIEHS will continue their efforts for the next 50 years and beyond to provide findings from basic and applied research to inform appropriate policy decisions to protect public health.
